# *APOE* ɛ4 carriership determines a faster plasma p‐tau217 progression in Aβ‐positive individuals

**DOI:** 10.1002/alz.71048

**Published:** 2025-12-26

**Authors:** Marcel S. Woo, Arthur C Macedo, Seyyed Ali Hosseini, Joseph Therriault, Yi‐Ting Wang, Nesrine Rahmouni, Étienne Aumont, Stijn Servaes, Cécile Tissot, Jaime Fernandez‐Arias, Lydia Trudel, Brandon Hall, Gleb Bezgin, Kely Quispialaya‐Socualaya, Marina Goncalves, Tevy Chan, Jenna Stevenson, Yansheng Zheng, Robert Hopewell, Stuart Mitchell, Firoza Z. Lussier, Alzheimer's Disease Neuroimaging Initiative (ADNI), Paolo Vitali, Gassan Massarweh, Jean‐Paul Soucy, Andréa L. Benedet, Serge Gauthier, Henrik Zetterberg, Kaj Blennow, Tharick A. Pascoal, Pedro Rosa‐Neto

**Affiliations:** ^1^ Translational Neuroimaging Laboratory, The McGill University Research Centre for Studies in Aging, McConnell Brain Imaging Centre (BIC) Montreal Neurological Institute Montréal Quebec Canada; ^2^ Translational Neurodegeneration Laboratory, Department of Neurology University Medical Centre Hamburg Eppendorf Hamburg Germany; ^3^ Institute of Neuroimmunology and Multiple Sclerosis University Medical Centre Hamburg Eppendorf Hamburg Germany; ^4^ Department of Neurology and Neurosurgery McGill University Montréal Quebec Canada; ^5^ Department of Molecular Biophysics and Integrated Bioimaging Lawrence Berkeley National Laboratory Berkeley California USA; ^6^ Department Psychiatry, School of Medicine University of Pittsburgh Pittsburgh Pennsylvania USA; ^7^ Department of Neurology, School of Medicine University of Pittsburgh Pittsburgh Pennsylvania USA; ^8^ Department of Psychiatry and Neurochemistry, Institute of Neuroscience and Physiology, The Sahlgrenska Academy University of Gothenburg Mölndal Sweden; ^9^ Clinical Neurochemistry Laboratory Sahlgrenska University Hospital Mölndal Sweden; ^10^ Department of Neurodegenerative Disease UCL Institute of Neurology London UK; ^11^ UK Dementia Research Institute at UCL London UK; ^12^ Hong Kong Center for Neurodegenerative Diseases Hong Kong China; ^13^ UW Department of Medicine, Wisconsin Alzheimer's Disease Research Center University of Wisconsin School of Medicine and Public Health, University of Wisconsin–Madison Madison Wisconsin USA; ^14^ Peter O'Donnell Jr. Brain Institute (OBI) Dallas Texas USA

**Keywords:** Alzheimer's disease, apolipoprotein E, biomarker, disease progression, phosphorylated tau217

## Abstract

**INTRODUCTION:**

It is unclear whether the different Alzheimer's disease (AD) progression trajectories of apolipoprotein E (*APOE*) ɛ4 carriers is reflected by blood phosphorylated tau (p‐tau) analytes.

**METHODS:**

We assessed longitudinal trajectories in plasma p‐tau181, 217, and 231, in amyloid beta–positive (A+) and negative (A−) *APOE* ɛ4 carriers (E+) or non‐carriers (E–). We included 2039 participants from the observational Translational Biomarkers in Aging and Dementia (TRIAD) and Alzheimer's Disease Neuroimaging Initiative cohorts, categorized into 840 A−E–, 251 A–E+, 386 A+E4−, and 616 A+E4+. Longitudinal data were available for 1045 participants.

**RESULTS:**

In TRIAD, ALZpath p‐tau217 (*β* = 0.45, *p* = 0.02) and p‐tau217+^Janssen^ (*β* = 0.67, *p* = 0.002), and in ADNI p‐tau217 (*β* = 0.90, *p* = 0.002) increased faster in A+E4+. This was not the case in E– or A– individuals or for p‐tau181 and p‐tau231.

**DISCUSSION:**

These findings suggest p‐tau217 as a marker of faster progression in *APOE* ɛ4 carriers, highlighting its potential in disease stratification.

**Highlights:**

- Blood phosphorylated tau (p‐tau)217 increases faster in apolipoprotein E (*APOE*) ɛ4 carriers with amyloid pathology.
- p‐tau181 and p‐tau231 do not increase faster in *APOE* ɛ4 carriers.
- *APOE* ɛ4 carriership does not change p‐tau in individuals without amyloid pathology.

## 1 BACKGROUND

Genome‐wide association studies identified apolipoprotein E (*APOE*)^1^ as the strongest genetic risk factor for Alzheimer's disease (AD). Homozygous *APOE* ɛ4 carriers have a 60% higher lifetime risk to develop AD dementia at the age of 85 years.^2^ Additionally, homozygous *APOE* ɛ4 carriers have a weaker treatment response to amyloid beta (Aβ)–removing therapies and are at higher risk for amyloid‐related imaging abnormalities (ARIAs).^3^, ^4^ Thus, a clearer understanding of AD disease progression in *APOE* ɛ4 carriers is an unmet clinical need.

*APOE* ɛ4 affects all hallmarks of AD: Aβ accumulation, tau accumulation, and neurodegeneration. Cross‐sectional studies using cerebrospinal fluid (CSF) biomarkers have shown that homozygous *APOE* ɛ4 carriers exhibit increased and earlier Aβ pathology.^5^ Additional positron emission tomography (PET) studies demonstrated that *APOE* ɛ4 is associated with tau accumulation independently of Aβ.^6^ Furthermore, longitudinal studies revealed that *APOE* ɛ4 amplifies the effect of Aβ accumulation on tau spreading and accumulation^7^ as well as age‐dependent microstructural changes of the brain.^8^ However, it is unclear whether these *APOE* ɛ4 dependent differences are similarly reflected by plasma biomarkers.

The discovery and ultra‐sensitive quantifications of different phosphorylated tau (p‐tau) analytes^9^, ^10^ allows the detection^11^, ^12^, ^13^ and staging^14^, ^15^ of AD pathology with similar accuracies in the CSF and plasma.^16^ Although PET‐based detection and staging^17^, ^18^ remains the in vivo gold standard, plasma biomarkers allow widely accessible quantifications of AD pathologies. P‐tau217 has emerged as an ultrasensitive biomarker to detect Aβ pathology that strongly improves the diagnostic accuracy for AD dementia in primary and secondary care.^19^, ^20^, ^21^ However, p‐tau217 not only reflects Aβ pathology but also shows a significant correlation with tau accumulation^22^, ^23^, ^24^ qualifying it as a biomarker for AD neuropathologic change. This study aimed to examine both cross‐sectional and longitudinal differences of plasma p‐tau analytes in participants with and without Aβ pathology (A+ and A–) measured by PET and CSF, stratified by *APOE* ɛ4 carriership. We hypothesized that *APOE* ɛ4 carriers with Aβ pathology have a faster increase of blood biomarkers compared to *APOE* ɛ4 non‐carriers or participants without Aβ pathology.

## 2 METHODS

### 2.1 Study participants

Participants of the Translational Biomarkers in Aging and Dementia (TRIAD) cohort signed consent forms before samples/data were collected. McGill University's ethics committee approved the study. The individuals that were enrolled in the TRIAD cohort^25^ underwent Aβ PET with [^18^F]AZD4694, tau PET with [^18^F]MK6240, and magnetic resonance imaging (MRI). The TRIAD cohort consists of a high proportion of individuals who were recruited from a specialized tertiary care memory clinic. The cognitively unimpaired individuals are adult volunteers from the community. Participants had a detailed clinical and cognitive assessment, including the Clinical Dementia Rating (CDR) and Mini‐Mental State Examination (MMSE). Cognitively unimpaired individuals had no objective cognitive impairment, a CDR score of 0, and were asked to report any subjective cognitive decline in a questionnaire given during screening. Individuals with mild cognitive impairment (MCI) had cognitive impairment, relatively preserved activities of daily living, and a CDR score of 0.5. Patients with mild‐to‐moderate AD clinical syndrome dementia had a CDR score between 0.5 and 2 and met the National Institute on Aging–Alzheimer's Association (NIA‐AA) criteria for probable AD determined by a dementia specialist.^25^, ^26^ Exclusion criteria for TRIAD were active substance abuse, recent head trauma, recent major surgery, or MRI/PET safety contraindications.^27^ All participants for whom the respective blood biomarkers, Aβ PET, and tau PET were available were included for this study. Furthermore, we included cognitively unimpaired, preclinical, and early AD subjects from the Alzheimer's Disease Neuroimaging Initiative (ADNI) database (adni.loni.usc.edu). The ADNI was launched in 2003 as a public–private partnership, led by Principal Investigator Michael W. Weiner, MD. The primary goal of ADNI has been to test whether serial MRI, PET, other biological markers, and clinical and neuropsychological assessment can be combined to measure the progression of MCI and AD. For up‐to‐date information, see www.adni‐info.org. The study was approved by the institutional review boards of all the participating institutions and informed written consent was obtained from all participants. Data used for the analyses presented here was accessed on January 31, 2025. We included participants for whom p‐tau217, p‐tau181, Aβ PET, and tau PET were available.

### 2.2 CSF data collection and analysis

For TRIAD, a standard protocol elaborated by McGill University Research Center for Studies was used to collect CSF samples during clinical evaluations. Written informed consent was provided by all participants. The CSF samples were aliquoted into polypropylene test tubes of 2 mL (0.5 mL minimum), centrifuged at 20°C, and frozen within 1 hour of the collection. CSF Aβ42 and Aβ40 were measured using the Lumipulse G1200. In ADNI, lumbar puncture was performed as described in the ADNI procedures manual (http://www.adni‐info.org/). CSF samples were frozen on dry ice within 1 hour after collection and shipped overnight on dry ice to the ADNI Biomarker Core laboratory at the University of Pennsylvania Medical Center. CSF Aβ1‐42 was measured using the Elecsys β‐amyloid(1‐42) assay.^28^

**RESEARCH IN CONTEXT**

1. **Systematic review**: By reviewing the literature in public databases and search engines, we identified the clinical need to identify fluid biomarkers that report faster disease progression in apolipoprotein E (*APOE*) ɛ4 carriers. No studies have evaluated longitudinal trajectories of phosphorylated tau (p‐tau) analytes in *APOE* ɛ4 carriers compared to *APOE* ɛ4 non‐carriers.
2. **Interpretation**: We find that p‐tau217 shows a faster increase in *APOE* ɛ4 carriers compared to *APOE* ɛ4 non‐carriers in two cohorts. This supports that p‐tau217 is the most sensitive biomarker to report disease progression and underlines the need to find therapeutic strategies for *APOE* ɛ4 carriers.
3. **Future directions**: Future studies should explore the molecular mechanisms linking *APOE* ɛ4 status to accelerated p‐tau217 accumulation. Additionally, clinical trials should evaluate whether *APOE* ɛ4‐targeted interventions can slow p‐tau217 progression and improve patient outcomes.

### 2.3 Plasma data collection and analysis

Plasma samples were collected at the screening visit, which generally precedes the first PET visit by ≈ 1 to 2 months according to standard procedures.^29^ Samples were then rapidly frozen for long‐term storage at −80°C.^29^ The Quanterix single molecule array (Simoa) HD‐X platform was used to quantify different tau species in plasma. p‐tau181 and p‐tau231 were quantified using in house–developed assays as previously described.^29^ Two commercially available p‐tau217 assays were evaluated: the Janssen R&D pTau‐217^+^ assay^30^ and the ALZpath pTau217 assay.^21^ The measurements of the different biomarkers have been described in detail elsewhere.^21^ One technical replicate was measured per sample. The following analytes were measured: pTau‐181, pTau‐231, pTau‐217^+^ Janssen, ALZpath pTau‐217. All plasma analytes were measured at the Department of Psychiatry and Neurochemistry, University of Gothenburg except for pTau‐217^+^ Janssen that was measured at Janssen R&D. All blood samples in ADNI were collected in ethylenediaminetetraacetic acid collection tubes and plasma was produced as described in the ADNI4 Procedures manual V2.0. The Lumipulse G1200 platform was used to measure p‐tau217. The Simoa Quanterix HD‐X platform was used to measure p‐tau181.

### 2.4 MRI acquisition and processing

Structural MRI data were acquired at the Montreal Neurological Institute (MNI) for all participants on a 3T Siemens Magnetom scanner using a standard head coil. Hippocampal volume was assessed using FreeSurfer version 6.0 and the Desikan–Killiany–Tourville atlas gray matter segmentation. As a measure of neurodegeneration, hippocampal volume was quantified with FreeSurfer version 5.1 in ADNI.

### 2.5 PET acquisition and processing

TRIAD participants were submitted to a T1‐weighted MRI, and [^18^F]AZD4694 and [^18^F]MK6240 PET scans. The PET scans were acquired using a brain‐dedicated Siemens high‐resolution research tomograph. [^18^F]MK6240 PET images were acquired at 90 to 110 minutes after the intravenous bolus injection of the radiotracer and reconstructed using an ordered subset expectation maximization algorithm on a 4D volume with four frames (4 × 300 seconds), as previously described.^31^ [^18^F]AZD4694 PET images were acquired at 40 to 70 minutes after the intravenous bolus injection of the radiotracer and reconstructed with the same ordered subset expectation maximization algorithm on a 4D volume with three frames (3 × 600 seconds).^25^ A 6 minute transmission scan with a rotating ^137^Cs point source was conducted at the end of each PET acquisition for attenuation correction. Images were corrected for motion, decay, dead time, and random and scattered coincidences. PET images were then linearly registered to the subject's T1‐weighted image space, and the T1‐weighted images were linearly and non‐linearly registered to the ADNI reference space. To minimize the influence of meningeal spillover into adjacent brain regions, [^18^F]MK6240 images were skull‐stripped in T1 space before transformations and blurring.^27^ The PET images in T1 space were linearly and non‐linearly registered to the ADNI space using transformations from the T1‐weighted image to ADNI space. [^18^F]MK6240 standardized uptake value ratios (SUVRs) were calculated using the inferior cerebellar gray matter as a reference region,^27^, ^32^ as derived from the SUIT cerebellum atlas.^33^ [^18^F]AZD4694 SUVRs were calculated using the whole cerebellum gray matter as the reference region. PET images were spatially smoothed to achieve an 8 mm full width at half‐maximum resolution. The global [^18^F]AZD4694 SUVR composite included the precuneus, prefrontal, orbitofrontal, parietal, temporal, and cingulate cortices.^33^

Detailed descriptions of ADNI neuroimaging acquisition and pre‐processing can be found elsewhere (http://adni.loni.usc.edu/datasamples/pet/). We used the neocortical [^18^F]Florbetapir SUVR that was normalized to the whole cerebellum. For tau PET, we analyzed the partial volume corrected [^18^F]AV1451 meta‐region of interest SUVR. The inferior cerebellar gray matter reference region using the SUIT template^34^ was used as reference. We only included data that passed the quality control.

### 2.6 Statistical analysis

All statistical analyses were performed within the R environment (version 4.4.1) using customized code. In TRIAD, participants with available Aβ PET were classified using a cut‐off of 1.55 [^18^F]AZD4694.^25^ If no Aβ PET was available, we classified participants with a CSF Aβ42/40 ratio < 0.068 as Aβ positive.^25^ For ADNI participants, a neocortical [^18^F]Florbetapir SUVR > 1.1^35^ or CSF Aβ1‐42 < 981^36^ were used as cut‐offs to determine Aβ positivity. Heterozygous and homozygous *APOE* ɛ4 carriers were summarized as *APOE* ɛ4 carriers in our statistical analyses. We used false discovery rate–corrected unpaired two‐tailed *t* tests for the statistical comparisons of the baseline values. For the longitudinal analyses, we calculated the differences of each parameter to the baseline value. Next, we fitted linear mixed effects models to estimate the effect of *APOE* ɛ4 carriership on the longitudinal progression of each biomarker in A+ and A– participants. We calculated the interaction between the time in years and *APOE* ɛ4 carriership. Covariates included age, sex, and the respective baseline value of each parameter. The participants were accounted for as random intercept in our model. To test whether the interactions between the covariates and the follow‐ups improved the model fit, we calculated the same longitudinal models including an interaction between follow‐up, the respective covariate, and *APOE* ɛ4 carriership. We then used an analysis of variance to compare the models with the covariate x follow‐up interaction with the model without this interaction. A full report of the standardized *β* values, 95% confidence intervals, and *p* values are provided in the tables and supporting information. The statistical test results are provided in the respective figure legends. *p* values < 0.05 were considered significant.

## 3 RESULTS

### 3.1 Study population in TRIAD

This study included 41 cognitively unimpaired young adults (CUY), 251 cognitively unimpaired (CU), 62 participants with MCI, 87 participants with AD dementia (ADD), and 113 participants with other neurological diseases (OND) from the TRIAD cohort. These were classified by Aβ status and *APOE* ɛ4 carriership. This resulted in 260 A– participants who were *APOE* ɛ4 non‐carriers (A– E–), 92 A– participants who were *APOE* ɛ4 carriers (A– E+; 8 homozygous), 92 A+ participants who were non‐carriers (A+ E–), and 110 A+ participants who were *APOE* ɛ4 carriers (A+ E+; 26 homozygous). The demographics for all TRIAD participants are available in Table 1. Longitudinal data were available for 103 A– E4– (mean follow‐up 21 months), 33 A– E4+ (mean follow‐up 22 months), 35 A+ E4– (mean follow‐up 22 months), and 45 A+ E4+ (mean follow‐up 19 months) participants. The demographics for participants with longitudinal follow‐ups are provided in Table S1 in supporting information.

**TABLE 1:** Demographics separated by amyloid status and *APOE* ɛ4 carriership in TRIAD.

Group	A– E4–	A– E4+	A+ E4–	A+ E4+
*N* (%)	260 (46.93)	92 (16.61)	92 (16.61)	110 (19.86)
Female, *N* (%)	166 (63.85)	49 (53.26)	54 (58.7)	62 (56.36)
Cognitively unimpaired young, *N* (%)	30 (11.54)	11 (11.96)	0 (0)	0 (0)
Cognitively unimpaired, *N* (%)	149 (57.31)	54 (58.7)	29 (31.52)	19 (17.27)
Mild cognitive impairment, *N* (%)	0 (0)	0 (0)	22 (23.91)	40 (36.36)
Alzheimer's disease dementia, *N* (%)	3 (1.15)	4 (4.35)	32 (34.78)	48 (43.64)
Other neurological disease, *N* (%)	78 (30)	23 (25)	9 (9.78)	3 (2.73)
*APOE* ɛ4 homozygous, *N*, (%)	0 (0)	8 (8.7)	0 (0)	26 (23.64)
Age, mean (SD)	62.42 (17.22)	59.27 (16.57)	70.43 (10.4)	68.81 (7.71)
MMSE, mean (SD)	28.53 (2.86)	29.06 (1.56)	26.24 (4.38)	25.01 (6.03)
[^18^F]AZD4694, SUVR, mean (SD)	1.23 (0.11)	1.25 (0.12)	2.24 (0.54)	2.33 (0.4)
[^18^F]MK6240, SUVR, mean (SD)	0.96 (0.31)	0.95 (0.2)	1.71 (1.02)	1.86 (0.84)
Hippocampal volume, mm3, mean (SD)	3806.37 (489.66)	3859.02 (499.36)	3532.86 (518.31)	3303.3 (540.6)
ALZpath p‐tau217, pg/mL, mean (SD)	0.28 (0.31)	0.28 (0.13)	1.01 (0.69)	1.1 (0.74)
p‐tau217+ Janssen, pg/mL, mean (SD)	0.06 (0.06)	0.05 (0.04)	0.17 (0.14)	0.21 (0.13)
p‐tau181, pg/mL, mean (SD)	7.74 (4.76)	8.5 (5.88)	12.74 (6.18)	12.38 (6.19)
p‐tau231, pg/mL, mean (SD)	13.06 (6.07)	14.92 (7.12)	21.73 (10.14)	20.38 (9.29)

Abbreviations: *APOE*, apolipoprotein E; E4–, *APOE* ɛ4 non‐carriers; E4+, *APOE* ɛ4 carriers; MMSE, Mini‐Mental State Examination; p‐tau, phosphorylated tau; SD, standard deviation; SUVR, standardized uptake value ratio; TRIAD, Translational Biomarkers in Aging and Dementia.

### 3.2 p‐tau217 increases faster in *APOE* ɛ4 carriers with amyloid accumulation in TRIAD

**FIGURE 1:**
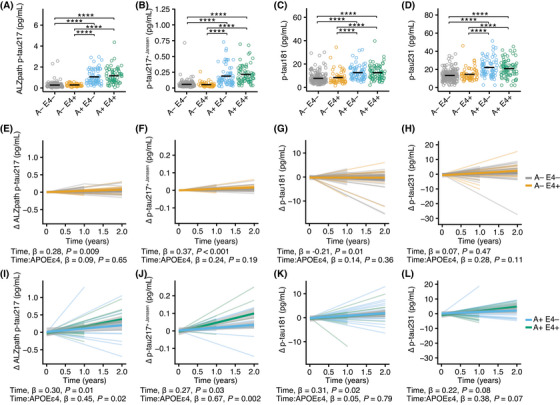
*APOE* ɛ4 carriers with Aβ accumulation have a faster p‐tau217 increase in TRIAD. A–D, Plasma levels of ALZpath p‐tau217 (A), p‐tau217^+ Janssen^ (B), p‐tau181 (C), and p‐tau231 (D) at the first visit in people with (A+) and without (A–) Aβ accumulation who are *APOE* ɛ4 carriers (A– E4+, *n* = 92; A+ E4+, *n* = 110) or non‐carriers (A– E4– *n* = 260; A+ E4– *n* = 92). Unpaired false discovery rate–adjusted *t* tests were used for statistical comparisons. ****p < 0.0001. E–H, Longitudinal changes of ALZpath p‐tau217 (E), p‐tau217^+ Janssen^ (F), p‐tau181 (G), and p‐tau231 (H) in participants without Aβ accumulation who are *APOE* ɛ4 non‐carriers (A– E4–; ALZpath p‐tau217, *n* = 44; p‐tau217^+ Janssen^, *n* = 47; p‐tau181, *n* = 51; p‐tau231, *n* = 48) or carriers (A– E4+; ALZpath p‐tau217, *n* = 21; p‐tau217^+ Janssen^, *n* = 20; p‐tau181, *n* = 24; p‐tau231, *n* = 21). I–L, Longitudinal changes of ALZpath p‐tau217 (I), p‐tau217^+ Janssen^ (J), p‐tau181 (K), and p‐tau231 (L) in participants with Aβ accumulation who are *APOE* ɛ4 non‐carriers (A+ E4–; ALZpath p‐tau217, *n* = 22; p‐tau217^+ Janssen^, *n* = 20; p‐tau181, *n* = 24; p‐tau231, *n* = 23) or carriers (A+ E4+; ALZpath p‐tau217, *n* = 22; p‐tau217^+ Janssen^, *n* = 18; p‐tau181, *n* = 22; p‐tau231, *n* = 19). Linear mixed effects models were used for statistical analyses. The standardized β‐estimates and *p* values for the time and interaction between time and *APOE* ɛ4 carriership is provided in the figure. The complete statistical results are shown in Tables 2 and 3. Aβ, amyloid beta; *APOE*, apolipoprotein E; p‐tau, phosphorylated tau; TRIAD, Translational Biomarkers in Aging and Dementia.

In a first step, we performed a cross‐sectional analysis and compared baseline Aβ and tau load measured by PET in A– E4– (*n* = 260), A– E4+ (*n* = 92), A+ E4– (*n* = 82), and A+ E4+ (*n* = 110) participants. While we confirmed higher Aβ (Figure S1A in supporting information) and tau PET (Figure S1B) levels in A+ participants, *APOE* ɛ4 carriers showed the same levels in both A– and A+ participants. Next, we compared the baseline plasma levels of ALZpath p‐tau217 (Figure 1A), p‐tau217^+ Janssen^ (Figure 1B), p‐tau181 (Figure 1C), and p‐tau231 (Figure 1D) in the same groups. Again, we did not detect any difference between *APOE* ɛ4 non‐carriers and carriers in A– and A+ participants. Because homozygous *APOE* ɛ4 carriers are particularly relevant to AD pathology, we compared the baseline ALZpath p‐tau217, p‐tau217^+ Janssen^, and p‐tau181 levels between heterozygous and homozygous *APOE* ɛ4 carriers. This revealed higher levels of all p‐tau analytes in homozygous compared to heterozygous *APOE* ɛ4 carriers (Figure S1C–F). Thus, while the baseline levels of Aβ and tau load as well as p‐tau181, 217, and 231 were not different among *APOE* ɛ4 carriers or non‐carriers in the TRIAD cohort, homozygous *APOE* ɛ4 carriers had significantly higher levels of the investigated p‐tau analytes than heterozygous *APOE* ɛ4 carriers.

In the next step, we analyzed whether Aβ and tau accumulation measured by PET or blood p‐tau biomarkers have different longitudinal trajectories in *APOE* ɛ4 carriers. To account for an attrition bias, we restricted our analysis in TRIAD to maximal 24 months follow‐up. The number of participants per time point and biomarker are shown in Table S2 in supporting information. First, we analyzed the longitudinal changes of ALZpath p‐tau217 (Figure 1E; E4– *n* = 44; E4+ *n* = 21), p‐tau217^+ Janssen^ (Figure 1F; E4– *n* = 47; E4+ *n* = 20), p‐tau181 (Figure 1G; E4– *n* = 51; E4+ *n* = 24), p‐tau231 (Figure 1H; E4– *n* = 48; E4+ *n* = 21), as well as Aβ accumulation (Figure S1G; E4– *n* = 85; E4+ *n* = 29) and tau accumulation measured by PET (Figure S1H; E4– *n* = 80; E4+ *n* = 27) in A– participants. Age, biological sex, and baseline values of the respective parameters were used as confounders in our mixed effect model. We detected an overall significant change of both p‐tau217 assays, p‐tau181, and Aβ PET over time. However, the interaction between time and *APOE* ɛ4 carriership did not reach significance for any of the PET or plasma biomarkers. Thus, *APOE* ɛ4 carriership did not impact Aβ or tau accumulation nor p‐tau181, 218, and 231 progressions in A– participants in our cohort. The statistical results for p‐tau181, 217, and 231 are provided in Table 2, and for Aβ and tau PET in Table S3 in supporting information.

**TABLE 2:** Results of longitudinal p‐tau181, 217, and 231 measurements in A– *APOE* ɛ4 carriers and non‐carriers in TRIAD.

Biomarker	Parameter	St. β	95% CI down	95% CI up	*p* value
ALZpath p‐tau217	Intercept	−0.029	−0.281	0.222	0.983
ALZpath p‐tau217	Time	0.275	0.071	0.478	0.009
ALZpath p‐tau217	*APOE* ɛ4 carriership	0.063	−0.376	0.502	0.968
ALZpath p‐tau217	Age	0.066	−0.117	0.25	0.477
ALZpath p‐tau217	Male sex	0.028	−0.387	0.442	0.895
ALZpath p‐tau217	Baseline	−0.152	−0.361	0.058	0.156
ALZpath p‐tau217	Time:*APOE* ɛ4 carriership	0.085	−0.284	0.454	0.65
p‐tau217^+ Janssen^	Intercept	−0.067	−0.292	0.158	0.69
p‐tau217^+ Janssen^	Time	0.373	0.184	0.563	< 0.001
p‐tau217^+ Janssen^	*APOE* ɛ4 carriership	0.08	−0.301	0.462	0.607
p‐tau217^+ Janssen^	Age	0.039	−0.127	0.206	0.642
p‐tau217^+ Janssen^	Male sex	0.125	−0.221	0.471	0.476
p‐tau217^+ Janssen^	Baseline	−0.01	−0.181	0.161	0.911
p‐tau217^+ Janssen^	Time:*APOE* ɛ4 carriership	0.241	−0.121	0.604	0.191
p‐tau181	Intercept	−0.044	−0.237	0.148	0.107
p‐tau181	Time	−0.212	−0.38	−0.043	0.014
p‐tau181	*APOE* ɛ4 carriership	0.225	−0.088	0.538	0.631
p‐tau181	Age	0.062	−0.082	0.207	0.396
p‐tau181	Male sex	−0.063	−0.365	0.239	0.681
p‐tau181	Baseline	−0.555	−0.698	−0.411	< 0.001
p‐tau181	Time:*APOE* ɛ4 carriership	0.142	−0.165	0.449	0.362
p‐tau231	Intercept	−0.037	−0.254	0.18	0.332
p‐tau231	Time	0.069	−0.12	0.258	0.47
p‐tau231	*APOE* ɛ4 carriership	0.193	−0.167	0.552	0.842
p‐tau231	Age	0.054	−0.107	0.215	0.509
p‐tau231	Male sex	−0.046	−0.386	0.295	0.791
p‐tau231	Baseline	−0.404	−0.567	−0.24	< 0.001
p‐tau231	Time:*APOE* ɛ4 carriership	0.277	−0.072	0.626	0.118

Abbreviations: *APOE*, apolipoprotein E; CI, confidence interval; E p‐tau, phosphorylated tau; St. β, standardized β estimate; TRIAD, Translational Biomarkers in Aging and Dementia.

Subsequently, we performed the same analyses in A+ participants. While our analysis revealed no significant overall increase in Aβ accumulation during the observed time period (Figure S1I; E4– *n* = 36; E4+ *n* = 42), tau accumulation (Figure S1J; E4– *n* = 31; E4+ *n* = 45), ALZpath p‐tau217 (Figure 1I; E4– *n* = 22; E4+ *n* = 22), p‐tau217^+ Janssen^ (Figure 1J; E4– *n* = 20; E4+ *n* = 18), and p‐tau181 (Figure 1K; E4– *n* = 24; E4+ *n* = 22) significantly increased in the follow‐up visits. The longitudinal p‐tau231 (Figure 1L; E4– *n* = 23; E4+ *n* = 19) analysis showed a similar trend but did not reach statistical significance. Notably, our data showed that *APOE* ɛ4 carriers had a faster increase of ALZpath p‐tau217 (*p* = 0.02, standardized *β* = 0.45) and p‐tau217^+ Janssen^ (*p* = 0.002, standardized *β* = 0.67) in contrast to p‐tau181, p‐tau231, and PET‐based quantifications of Aβ or tau accumulation. We concluded that longitudinal p‐tau217 was impacted by *APOE* ɛ4 carriership in A+ but not A– participants. The statistical results of A+ participants for p‐tau181, 217, and 231 are provided in Table 3, and for Aβ or tau PET in Table S3.

**TABLE 3:** Results of longitudinal p‐tau181, 217, and 231 measurements in A+ *APOE* ɛ4 carriers and non‐carriers in TRIAD.

Biomarker	Parameter	St. β	95% CI down	95% CI up	*p* value
ALZpath p‐tau217	Intercept	−0.257	−0.559	0.045	0.139
ALZpath p‐tau217	Time	0.303	0.062	0.544	0.015
ALZpath p‐tau217	*APOE* ɛ4 carriership	0.27	−0.098	0.639	0.639
ALZpath p‐tau217	Age	−0.163	−0.367	0.042	0.117
ALZpath p‐tau217	Male sex	0.341	−0.039	0.72	0.078
ALZpath p‐tau217	Baseline	−0.139	−0.341	0.064	0.177
ALZpath p‐tau217	Time:*APOE* ɛ4 carriership	0.451	0.074	0.828	0.02
p‐tau217^+ Janssen^	Intercept	−0.175	−0.477	0.127	0.084
p‐tau217^+ Janssen^	Time	0.267	0.026	0.507	0.031
p‐tau217^+ Janssen^	*APOE* ɛ4 carriership	0.379	−0.009	0.766	0.491
p‐tau217^+ Janssen^	Age	‐–0.174	−0.378	0.03	0.098
p‐tau217^+ Janssen^	Male sex	0.03	−0.379	0.439	0.884
p‐tau217^+ Janssen^	Baseline	−0.108	−0.311	0.096	0.302
p‐tau217^+ Janssen^	Time:*APOE* ɛ4 carriership	0.667	0.263	1.071	0.002
p‐tau181	Intercept	0.01	−0.33	0.351	0.041
p‐tau181	Time	0.305	0.044	0.566	0.023
p‐tau181	*APOE* ɛ4 carriership	−0.05	−0.462	0.362	0.724
p‐tau181	Age	−0.215	−0.428	−0.003	0.047
p‐tau181	Male sex	0.033	−0.386	0.452	0.877
p‐tau181	Baseline	−0.145	−0.355	0.066	0.176
p‐tau181	Time:*APOE* ɛ4 carriership	0.054	−0.364	0.473	0.797
p‐tau231	Intercept	−0.125	−0.446	0.196	0.15
p‐tau231	Time	0.222	−0.029	0.473	0.082
p‐tau231	*APOE* ɛ4 carriership	0.11	−0.29	0.511	0.425
p‐tau231	Age	−0.108	−0.326	0.11	0.326
p‐tau231	Male sex	0.201	−0.209	0.612	0.331
p‐tau231	Baseline	−0.319	−0.535	−0.103	0.004
p‐tau231	Time:*APOE* ɛ4 carriership	0.378	−0.033	0.79	0.071

Abbreviations: *APOE*, apolipoprotein E; CI, confidence interval; p‐tau = phosphorylated tau; St. β, standardized β estimate; TRIAD, Translational Biomarkers in Aging and Dementia.

Next, we tested whether interactions between age, sex, or the respective baseline values and time significantly affected the longitudinal trajectories. In A– participants, none of these interactions affected the longitudinal trajectories. In A+ participants, accounting for the interaction between age and time significantly improved the ALZpath p‐tau217 (*p* = 0.02), p‐tau217^+ Janssen^ (*p* < 0.001), and p‐tau181 (*p* = 0.004) models, accounting for the interaction between sex and time significantly improved the ALZpath p‐tau217 model fit (*p* = 0.04), and accounting for the interaction between the baseline values and time improved the ALZpath p‐tau217 model fit (*p* = 0.04). These findings indicate that in A+ individuals, the longitudinal increase in p‐tau levels varies with age, sex, and baseline biomarker concentration, while such moderation effects were not observed in A− individuals.

### 3.3 Study population in ADNI

To replicate our findings in an independent cohort, we included 1539 ADNI participants that encompassed 614 CN, 646 people with MCI, 240 people with ADD, and 39 people with OND. These were divided into 580 A– E4–, 160 A– E4+ (11 homozygous carriers), 293 A+ E4– and 506 A+ E4+ (118 homozygous carriers). The demographics for all ADNI participants separated by Aβ status and *APOE* ɛ4 carriership are provided in Table 4. Longitudinal data were available for 328 A– E4– (mean follow‐up 3.9 years), 85 A– E4+ (mean follow‐up 3.5 years), 162 A+ E4– (mean follow‐up 3.8 years), and 254 A+ E4+ (mean follow‐up 3.5 years). The demographics for participants with longitudinal follow‐ups are provided in Table S4 in supporting information.

**TABLE 4:** Demographics separated by amyloid status and *APOE* ɛ4 carriership in ADNI.

Group	A– E4–	A– E4+	A+ E4–	A+ E4+
*N* (%)	580 (37.69)	160 (10.4)	293 (19.04)	506 (32.88)
Female, *N* (%)	274 (47.24)	74 (46.25)	138 (47.1)	245 (48.42)
Cognitively unimpaired, *N* (%)	317 (54.66)	89 (55.62)	109 (37.2)	99 (19.57)
Mild cognitive impairment, *N* (%)	217 (37.41)	66 (41.25)	121 (41.3)	242 (47.83)
Alzheimer's disease dementia, *N* (%)	29 (5)	2 (1.25)	55 (18.77)	154 (30.43)
Other neurological disease, *N* (%)	17 (2.93)	3 (1.88)	8 (2.73)	11 (2.17)
*APOE* ɛ4 homozygous, *N*, (%)	0 (0)	11 (6.88)	0 (0)	118 (23.32)
Age, mean (SD)	72.07 (7.19)	68.97 (6.91)	75.3 (6.94)	72.51 (6.85)
MMSE, mean (SD)	28.53 (2.07)	28.76 (1.35)	27.31 (2.77)	26.18 (3.55)
[^18^F]Florbetapir, SUVR, mean (SD)	1 (0.05)	1.02 (0.05)	1.34 (0.18)	1.4 (0.17)
[^18^F]AV1451, SUVR, mean (SD)	1.22 (0.16)	1.18 (0.11)	1.5 (0.51)	1.78 (0.69)
CSF Aβ1‐42, pg/mL, mean (SD)	1407.52 (325.63)	1297.32 (377.34)	849.69 (359.83)	669.73 (256.1)
p‐tau217, pg/mL, mean (SD)	0.14 (0.19)	0.12 (0.09)	0.37 (0.3)	0.51 (0.38)
p‐tau181, pg/mL, mean (SD)	14.44 (11.8)	14.78 (10.76)	19.93 (12.3)	22.73 (9.97)

Abbreviations: Aβ, amyloid beta; ADNI, Alzheimer's Disease Neuroimaging Initiative; *APOE*, apolipoprotein E; CSF, cerebrospinal fluid; E4–, *APOE* ɛ4 non‐carriers; E4+, *APOE* ɛ4 carriers; MMSE, Mini‐Mental State Examination; p‐tau, phosphorylated tau; SD, standard deviation; SUVR, standardized uptake value ratio.

### 3.4 p‐tau217 increases faster in *APOE* ɛ4 carriers with amyloid accumulation in ADNI

**FIGURE 2:**
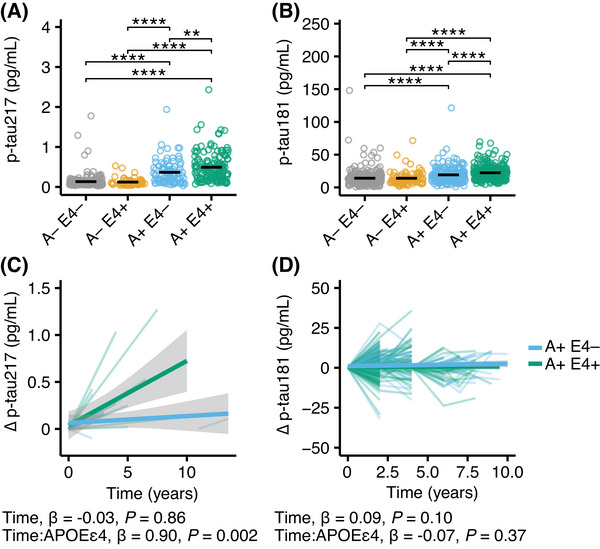
*APOE* ɛ4 carriers with Aβ accumulation have a faster p‐tau217 increase in ADNI. A,B, Plasma levels of p‐tau217 (A), and p‐tau181 (B) at the first visit in people with (A+) and without (A–) Aβ accumulation who are *APOE* ɛ4 non‐carriers (p‐tau217, A– E4– *n* = 211; A+ E4– *n* = 89; p‐tau181, A– E4– *n* = 303; A+ E4– *n* = 176) or carriers (p‐tau217, A– E4+, *n* = 56; A+ E4+, *n* = 148; p‐tau181, A– E4+, *n* = 85; A+ E4+, *n* = 306). Unpaired false discovery rate–adjusted *t* tests were used for statistical comparisons. **p < 0.01, ****p < 0.0001. C,D, Longitudinal changes of p‐tau217 (C), and p‐tau181 (D) in participants with Aβ accumulation who are *APOE* ɛ4 non‐carriers (A+ E4–; p‐tau217, *n* = 8; p‐tau181, *n* = 127) or carriers (A+ E4+; p‐tau217, *n* = 12; p‐tau181, *n* = 200). Linear mixed effects models were used for statistical analyses. The standardized β estimates and *p* values for the time and interaction between time and *APOE* ɛ4 carriership is provided in the figure. The complete statistical results are shown in Table 5. Aβ, amyloid beta; ADNI, Alzheimer's Disease Neuroimaging Initiative; *APOE*, apolipoprotein E; p‐tau, phosphorylated tau.

First, we performed a cross‐sectional analysis of the baseline Aβ PET (Figure S2A in supporting information), tau PET (Figure S2B), plasma p‐tau217 (Figure 2A), and p‐tau181 (Figure 2B) values. As expected, these were increased in A+ participants compared to A–. Additionally, A+ E4+ showed higher baseline Aβ PET, tau PET, plasma p‐tau181, and plasma p‐tau217 compared to A+ E4–, which was not the case for A– participants. The plasma levels of p‐tau217 and p‐tau181 were higher in homozygous *APOE* ɛ4 carriers compared to heterozygous *APOE* ɛ4 carriers (Figure S2C,D).

In the next step, we analyzed the longitudinal progression of Aβ and tau accumulation measured by PET or the plasma p‐tau biomarkers of *APOE* ɛ4 carriers compared to non‐carriers. Similar to what we found in TRIAD, we did not find any differences of longitudinal Aβ accumulation (Figure S2E; E4– *n* = 272; E4+ *n* = 61), tau accumulation (Figure S2F; E4– *n* = 39; E4+ *n* = 12), and p‐tau181 (Figure S2G; E4– *n* = 264; E4+ *n* = 58) in A– E4+ compared to A– E4–. The limited sample size did not allow us to analyze p‐tau217 in A– (E4– *n* = 7; E4+ *n* = 1) or restrict the follow‐up time points. The number of participants per time point and biomarker are shown in Table S5 in supporting information. The statistical results for p‐tau181, Aβ, or tau PET in A– participants are provided in Table S6 in supporting information.

Next, we performed the same analyses in A+ participants. We detected an overall significant longitudinal increase of Aβ (E4– *n* = 135; E4+ *n* = 214), and tau accumulation (E4– *n* = 24; E4+ *n* = 35) measured by PET in this subset. Comparing A+ E4+ to A+ E4– revealed a significantly faster increase of p‐tau217 (Figure 2C; E4– *n* = 8; E4+ *n* = 12; *p* = 0.002, standardized *β* = 0.90) but not p‐tau181 (Figure 2D; E4– *n* = 127; E4+ *n* = 200), Aβ (Figure S2H), and tau accumulation (Figure S2I) measured by PET. The statistical results for p‐tau217 and p‐tau181 are provided in Table 5, and for Aβ and tau accumulation in Table S6.

**TABLE 5 alz71048-tbl-0005:** Results of longitudinal p‐tau181 and 217 measurements in A+ *APOE* ɛ4 carriers and non‐carriers in ADNI.

Biomarker	Parameter	St. β	95% CI down	95% CI up	*p* value
p‐tau217	Intercept	0.059	−0.609	0.727	0.774
p‐tau217	Time	−0.032	−0.403	0.339	0.862
p‐tau217	*APOE* ɛ4 carriership	0.679	−0.009	1.366	0.992
p‐tau217	Age	0.105	−0.25	0.459	0.551
p‐tau217	Male sex	−0.632	−1.253	−0.011	0.046
p‐tau217	Baseline	0.078	−0.202	0.359	0.572
p‐tau217	Time:*APOE* ɛ4 carriership	0.899	0.358	1.44	0.002
p‐tau181	Intercept	−0.036	−0.178	0.107	0.838
p‐tau181	Time	0.09	−0.018	0.199	0.103
p‐tau181	*APOE* ɛ4 carriership	−0.009	−0.17	0.152	0.613
p‐tau181	Age	0.048	−0.03	0.126	0.23
p‐tau181	Male sex	0.076	−0.075	0.227	0.325
p‐tau181	Baseline	−0.306	−0.383	−0.228	< 0.001
p‐tau181	Time:*APOE* ɛ4 carriership	−0.066	−0.21	0.079	0.373

Abbreviations: ADNI, Alzheimer's Disease Neuroimaging Initiative; *APOE*, apolipoprotein E; CI, confidence interval; p‐tau = phosphorylated tau; St. β, standardized β estimate.

Finally, we tested whether the interaction between the covariates and the follow‐ups significantly impacted our longitudinal models in ADNI. In A– participants, this was the not case for age and sex but including the interaction between the respective baseline values and the follow‐ups improved the models for p‐tau217 (*p* < 0.001) and p‐tau181 (*p* < 0.001). In A+ participants, accounting for the interaction between sex and time significantly improved the model fit for p‐tau217 (*p* < 0.001), and accounting for the interaction between the baseline value and time significantly improved the p‐tau181 model fit (*p* < 0.0001) while the interaction between age and the follow‐ups had no significant effects.

## 4 DISCUSSION

This study evaluated the cross‐sectional and longitudinal differences of Aβ and tau accumulation measured by PET and plasma p‐tau181, 217, and 231 in A+ and A– *APOE* ɛ4 non‐carriers compared to *APOE* ɛ4 carriers in two separate cohorts. We identified that only *APOE* ɛ4 carriers with abnormal Aβ pathology showed a faster p‐tau217 increase. Notably, this was found with three separate p‐tau217 assays; namely, ALZpath p‐tau217, p‐tau217^+Janssen^, and C2N p‐tau217, excluding assay‐specific biases. Additionally, our results showed that the faster p‐tau217 increase required the presence of Aβ pathology, because we did not observe this effect in A– participants. This underlines the specificity of the *APOE* ɛ4 effect on the longitudinal plasma p‐tau217 changes in the AD spectrum.

Faster p‐tau217 pathophysiological progression found in this study is in line with imaging and biomarker studies in large human AD continuum cohorts that demonstrated an association between *APOE* ɛ4 and both tau accumulation and spreading.^6^, ^7^, ^37^ Similarly, animal literature demonstrated that a human *APOE* ɛ4 knock‐in exacerbates tauopathy‐associated neurodegeneration.^38^ Several mechanisms have been identified that might explain the faster disease progression in *APOE* ɛ4 carriers. *APOE* ɛ4 impairs the lipid shuttling in the brain and thereby leads to a neuronal energy deficiency.^39^, ^40^ Furthermore, the impaired lipid transport induces lipid droplet accumulation in glia cells, leaving them dysfunctional.^41^, ^42^, ^43^ Particularly, microglia that accumulate lipid droplets have a reduced phagocytic activity and secrete neurotoxic factors, allowing the accumulation of Aβ, tau spread, and neuronal loss.^41^, ^42^, ^43^ Thus, our findings are well in line with mechanistic studies that observed an accelerated disease progression in *APOE* ɛ4 carriers or AD mouse models that investigated the impact of *APOE* ɛ4. The faster increase in p‐tau 217 compared to other fragments might point toward the highest sensitivity of p‐tau217 to monitor disease progression.^44^

*APOE* ɛ4 is also associated with higher vascular burden.^45^ On the one hand, *APOE* ɛ4 carriers probably have a higher prevalence of cerebral amyloid angiopathy with vascular Aβ accumulation.^46^ On the other hand, *APOE* ɛ4 accelerates vascular dysfunction independent of Aβ in AD mouse models.^47^ Similar to *APOE* ɛ4,^7^ vascular burden potentiates the effect of Aβ on tau accumulation and disease progression^48^ and has been recognized as an important contributor to AD progression in the latest criteria for diagnosis and staging of AD by the Alzheimer's Association.^49^ The association between *APOE* ɛ4 and vascular pathologies is particularly relevant for Aβ‐targeting therapies because homozygous *APOE* ɛ4 carriers have a higher incidence of ARIAs with currently available anti‐amyloid monoclonal antibodies.^3^, ^4^ Given the faster and earlier disease progression of *APOE* ɛ4 carriers that we and others^5^ observed, *APOE* ɛ4 homozygotes are a particularly vulnerable group that requires disease‐modifying treatments. Estimating the disease dynamics by longitudinal p‐tau217 measurements may help to identify *APOE* ɛ4 carriers who will likely benefit from Aβ‐removing therapies. Further research is warranted to investigate the interaction among *APOE* ɛ4, vascular burden, and Aβ‐removing therapies to identify the mechanisms behind ARIAs and find new biomarkers and potential mitigating and preventive strategies for ARIAs.[Table alz71048-tbl-0005]

The differences in disease progression between *APOE* ɛ4 carriers and non‐carriers were only reflected by the faster increase of p‐tau217. As expected, Aβ and tau accumulation, as well as all p‐tau analytes, significantly increased over time in A+ participants. However, we did not observe any *APOE* ɛ4–dependent differences in the progression of PET Aβ or PET tau accumulation, plasma p‐tau181, or plasma p‐tau231. This suggests that in addition to Aβ pathology,^19^, ^50^, ^51^, ^52^, ^53^ different p‐tau analytes may indicate specific, different disease pathways. Indeed, this is supported by a histopathological study that investigated the subcellular localization of different p‐tau variants in *post mortem* brains of deceased people with AD.^54^ The same study showed a strong co‐localization of p‐tau181, 217, and 231 in neurofibrillary tangles and neuropil threads. However, only p‐tau217 was found in multivesicular bodies and granulovacuolar degeneration bodies, which are neuron‐specific vacuolar bodies that are induced by neurofibrillary tangles.^54^ Furthermore, only p‐tau217 showed a significant overall increase in A– participants, which was not different in *APOE* ɛ4 carriers. This is in line with studies that demonstrated a high association between p‐tau217 and subthreshold Aβ load.^20^ Along these lines, electron microscopy studies in aged macaques showed trans‐synaptic trafficking of p‐tau217^55^ explaining how p‐tau217 can be measured in body fluids before any structural or integral neuronal damage and they showed that the trans‐synaptic p‐tau217 signaling increases during aging. It is tempting to speculate that abnormal Aβ leads to a loss of the controlled tau phosphorylation and therefore gives rise to the different p‐tau analytes that are highly correlated in the pathological state. Additional histopathology and functional studies are required to further delineate the specific pathways that lead to p‐tau181, 231, and 217 phosphorylation and how they are promoted in *APOE* ɛ4 carriers.

The faster p‐tau217 increase in *APOE* ɛ4 carriers is in accordance with the accelerated cognitive decline of *APOE* ɛ4 carriers, supporting the notion that p‐tau217 is superior to the other p‐tau analytes to measure disease progression.^44^ This notion is further supported by studies showing that plasma p‐tau217 identifies cognitively normal older adults who will develop a cognitive impairment in the near future^56^, ^57^ and informs about the dementia risk in people with MCI.^58^ Thus, p‐tau217 not only detects AD pathology but also is a prognostic biomarker for monitoring disease progression.^59^

Our findings should be carefully interpreted given certain methodological limitations. While our cross‐sectional analysis revealed higher p‐tau217 and p‐tau181 levels in homozygous compared to heterozygous *APOE* ɛ4 carriers, the limited longitudinal data did not allow separate analyses of heterozygous and homozygous *APOE* ɛ4 carriers to test for gene–dose effects, which is of high clinical interest for disease prognosis, patient enrichment for clinical trials, and Aβ‐removing antibodies. Although we separated A+ and A– participants in our analyses, the limited sample size did not allow further separation by diagnosis or cognitive impairment status. Thus, we cannot conclude whether the differences in p‐tau217 progression already happen in presymptomatic or mildly affected individuals with Aβ pathology. Larger sample sizes are required to test whether the p‐tau217 progression is similar in A+ individuals with or without cognitive impairment. In addition, this study does not offer any mechanistic explanations for the specifically faster p‐tau217 progression in *APOE* ɛ4 carriers. Our data support the notion that specific pathways are induced by different tau phosphorylation sites^54^, ^55^ and therefore might report about distinct pathologies in addition to Aβ accumulation. This needs to be addressed by further mechanistic studies. Last, we observed a considerable variability of the individual trajectories over time reflecting the relatively low dynamic range of plasma p‐tau measures, analytical variation across visits, and potential biological fluctuations, potentially driven by age, sex, circadian, metabolic, or vascular factors. Notably, including the interaction between age/sex and the follow‐ups improved the model fit for some p‐tau analytes highlighting the biological variability of p‐tau trajectories. Although our mixed‐effects models accounted for within‐subject variability by including random intercepts and thus allowed estimation of group‐level effects across multiple time points, this variability remains a key limitation of blood‐based p‐tau analytes for individual‐level prediction. Further methodological optimization and standardization are needed to improve the interpretation of longitudinal p‐tau data in both clinical trials and clinical practice.

Despite these limitations, our study highlights that *APOE* ɛ4 carriers have a faster plasma p‐tau217 increase reflecting their faster disease progression. Thus, our study supports that p‐tau217 is the most sensitive biomarker to measure disease progression in AD. Detecting different longitudinal p‐tau217 trajectories will be beneficial as potential endpoints and patient enrichment in clinical trials as well as treatment monitoring. *APOE* ɛ4 carriership still poses a major challenge, and a better understanding of *APOE* biology might lead to the development of novel disease‐modifying therapies for people with AD.

## CONFLICT OF INTEREST STATEMENT

M.S. Woo received honoraria from Lilly for educational lectures. J. Therriault has served as a consultant for the Neurotorium educational platform and for Alzheon Inc. P. Vitali serves on the scientific advisory boards for NovoNordisc, Eisai, and Lilly, and received honoraria from IntelGenx Corp. S. Gauthier serves on scientific advisory boards for Alzeon, AmyriAD, Advantage, Eisai Canada, Enigma USA, Lilly Canada, Medesis, Lundbeck Foundation, Novo‐Nordisk Canada, Okutsa, and TauRx. H. Zetterberg serves on the scientific advisory boards and/or as a consultant for Abbvie, Acumen, Alector, Alzinova, ALZPath, Amylyx, Annexon, Apellis, Artery Therapeutics, AZTherapies, Cognito Therapeutics, CogRx, Denali, Eisai, Merry Life, Nervgen, Novo Nordisk, Optoceutics, Passage Bio, Pinteon Therapeutics, Prothena, Red Abbey Labs, reMYND, Roche, Samumed, Siemens Healthineers, Triplet Therapeutics, and Wave; has given lectures in symposia sponsored by Alzecure, Biogen, Cellectricon, Fujirebio, Lilly, Novo Nordisk, and Roche; and is a cofounder of Brain Biomarker Solutions in Gothenburg AB (BBS), which is a part of the GU Ventures Incubator Program (outside submitted work). B.J. Jean‐Claude reports no disclosures relevant to the manuscript. K. Blennow reports having served as a consultant and on advisory boards for AC Immune, Acumen, ALZPath, AriBio, BioArctic, Biogen, Eisai, Lilly, Moleac Pte. Ltd, Novartis, Ono Pharma, Prothena, Roche Diagnostics, and Siemens Healthineers; has served on data monitoring committees for Julius Clinical and Novartis; has given lectures, produced educational materials, and participated in educational programs for AC Immune, Biogen, Celdara Medical, Eisai, and Roche Diagnostics; and is a co‐founder of Brain Biomarker Solutions in Gothenburg AB (BBS), which is a part of the GU Ventures Incubator Program, outside the work presented in this paper. P. Rosa‐Neto reports no disclosures relevant to the manuscript. Y.T. Wang, S.A. Hossein, A.C. Macedo, N.R. Rahmouni, E. Aumont, S. Servaes, C. Tissot, J. Fernandez‐Arias, L. Trudel, B. Hall, G. Bezgin, K. Quispialaya‐Socualaya, M. Goncalves, T. Chan, J. Stevenson, Y. Zheng, F.Z. Lussier, G. Massarweh, J.P. Soucy, A.L. Benedet, T.A. Pascoal report no disclosures relevant to the manuscript.

## CONSENT STATEMENT

Participants of the Translational Biomarkers in Aging and Dementia (TRIAD) cohort signed consent forms before samples/data were collected. McGill University's ethics committee approved the study. The ADNI study was approved by the institutional review boards of all the participating institutions and informed written consent was obtained from all participants.

## Supporting information



Supporting Information

Supporting Information
