# Supplementary material for: APOE ɛ4 carriership determines a faster plasma p‐tau217 progression in Aβ‐positive individuals
Source: Alzheimers Dement. 2025 Dec 26;21(12):e71048. doi: 10.1002/alz.71048 (PMC12741900; doi:10.1002/alz.71048)
Supplement: Supplementary file 2 — Supporting Information [file ALZ-21-e71048-s001.docx]

**Supplementary Figures and Tables**

**Table S1. Demographics of all participants with longitudinal data in TRIAD.**

Abbreviations: CI = Cognitively impaired, E4- = APOE ɛ4 non-carriers, E4+ = APOE ɛ4 carriers, SD = Standard deviation, SUVR = Standard uptake value ratio.

| Group | A- E4- | A- E4+ | A+ E4- | A+ E4+ |
| --- | --- | --- | --- | --- |
| N (%) | 103 (47.69) | 33 (15.28) | 35 (16.2) | 45 (20.83) |
| Follow-up, months, mean (SD) | 20.97 (5.24) | 22.18 (4.37) | 21.6 (4.87) | 19.47 (5.88) |
| Female, N (%) | 67 (65.05) | 18 (54.55) | 19 (54.29) | 24 (53.33) |
| Cognitively unimpaired young, N (%) | 13 (12.62) | 1 (3.03) | 0 (0) | 0 (0) |
| Cognitively unimpaired, N (%) | 69 (66.99) | 24 (72.73) | 14 (40) | 9 (20) |
| Mild cognitive impairment, N (%) | 0 (0) | 0 (0) | 10 (28.57) | 20 (44.44) |
| Alzheimer's dementia, N (%) | 0 (0) | 2 (6.06) | 11 (31.43) | 16 (35.56) |
| Other neurological disease, N (%) | 21 (20.39) | 6 (18.18) | 0 (0) | 0 (0) |
| APOE ɛ4 homozygous, N, (%) | 0 (0) | 4 (12.12) | 0 (0) | 11 (24.44) |
| Age, mean (SD) | 62.53 (17.23) | 63.88 (12.4) | 68.8 (10.24) | 69.38 (6.97) |
| MMSE, mean (SD) | 28.94 (1.73) | 29.03 (1.88) | 27.12 (3.17) | 26 (5.3) |
| [^18^F]AZD4694, SUVR, mean (SD) | 1.25 (0.11) | 1.29 (0.11) | 2.15 (0.43) | 2.3 (0.36) |
| [^18^F]MK6240, SUVR, mean (SD) | 0.92 (0.1) | 1 (0.28) | 1.41 (0.64) | 1.77 (0.76) |
| Hippocampal volume, mm3, mean (SD) | 3861.41 (481.97) | 3712.72 (485.36) | 3626.54 (509.77) | 3339.86 (497.38) |
| ALZpath p-tau217, pg/mL, mean (SD) | 0.21 (0.11) | 0.3 (0.13) | 0.95 (0.6) | 1.05 (0.62) |
| p-tau217+ Janssen, pg/mL, mean (SD) | 0.04 (0.02) | 0.06 (0.05) | 0.16 (0.15) | 0.17 (0.1) |
| p-tau181, pg/mL, mean (SD) | 6.61 (3.49) | 7.69 (3.87) | 11.74 (5.91) | 11.09 (4.53) |
| p-tau231, pg/mL, mean (SD) | 11.47 (5.26) | 13.94 (5.1) | 19.22 (9.85) | 19.29 (7.58) |

**Table S2. Number of longitudinal TRIAD participants per timepoint.**

| **Amyloid status** | **Biomarker** | **Baseline** | **Year 1** | **Year 2** |
| --- | --- | --- | --- | --- |
| A+ | ALZpath p-tau217 | 45 | 16 | 24 |
| A+ | p-tau217+ Janssen | 39 | 17 | 17 |
| A+ | p-tau181 | 47 | 17 | 25 |
| A+ | p-tau231 | 43 | 16 | 23 |
| A+ | [^18^F]AZD4694 | 74 | 19 | 61 |
| A+ | [^18^F]MK6240 | 69 | 19 | 59 |
| A- | ALZpath p-tau217 | 64 | 22 | 42 |
| A- | p-tau217+ Janssen | 67 | 22 | 43 |
| A- | p-tau181 | 73 | 24 | 51 |
| A- | p-tau231 | 68 | 23 | 46 |
| A- | [^18^F]AZD4694 | 117 | 22 | 88 |
| A- | [^18^F]MK6240 | 109 | 25 | 81 |

**Table S3. Results of longitudinal amyloid and tau PET measurements in A- and A+ APOE ɛ4 carriers and non-carriers in TRIAD.**

Abbreviations: CI = Confidence interval, p-tau = phospho-tau, St. β = Standardized β-estimate.

| Amyloid status | Biomarker | Parameter | St. β | 95% CI down | 95% CI up | *P*-value |
| --- | --- | --- | --- | --- | --- | --- |
| A- | [^18^F]AZD4694 | Intercept | -0.038 | -0.219 | 0.143 | 0.031 |
| A- | [^18^F]AZD4694 | Time | -0.124 | -0.27 | 0.022 | 0.096 |
| A- | [^18^F]AZD4694 | APOE ɛ4 carriership | 0.257 | -0.058 | 0.572 | 0.618 |
| A- | [^18^F]AZD4694 | Age | 0.035 | -0.116 | 0.187 | 0.646 |
| A- | [^18^F]AZD4694 | Male sex | -0.07 | -0.353 | 0.214 | 0.63 |
| A- | [^18^F]AZD4694 | Baseline | -0.161 | -0.312 | -0.009 | 0.038 |
| A- | [^18^F]AZD4694 | Time:APOE ɛ4 carriership | 0.164 | -0.123 | 0.452 | 0.262 |
| A- | [^18^F]MK6240 | Intercept | 0.038 | -0.151 | 0.227 | < 0.001 |
| A- | [^18^F]MK6240 | Time | -0.019 | -0.09 | 0.051 | 0.586 |
| A- | [^18^F]MK6240 | APOE ɛ4 carriership | 0.211 | -0.118 | 0.541 | 0.259 |
| A- | [^18^F]MK6240 | Age | -0.047 | -0.184 | 0.091 | 0.504 |
| A- | [^18^F]MK6240 | Male sex | -0.26 | -0.542 | 0.023 | 0.073 |
| A- | [^18^F]MK6240 | Baseline | -0.883 | -1.009 | -0.758 | < 0.001 |
| A- | [^18^F]MK6240 | Time:APOE ɛ4 carriership | 0.008 | -0.13 | 0.147 | 0.906 |
| A+ | [^18^F]AZD4694 | Intercept | 0.078 | -0.176 | 0.332 | 0.011 |
| A+ | [^18^F]AZD4694 | Time | 0.138 | -0.076 | 0.351 | 0.204 |
| A+ | [^18^F]AZD4694 | APOE ɛ4 carriership | 0.069 | -0.231 | 0.369 | 0.393 |
| A+ | [^18^F]AZD4694 | Age | 0.006 | -0.144 | 0.157 | 0.936 |
| A+ | [^18^F]AZD4694 | Male sex | -0.277 | -0.578 | 0.023 | 0.07 |
| A+ | [^18^F]AZD4694 | Baseline | -0.358 | -0.51 | -0.205 | < 0.001 |
| A+ | [^18^F]AZD4694 | Time:APOE ɛ4 carriership | -0.115 | -0.412 | 0.183 | 0.449 |
| A+ | [^18^F]MK6240 | Intercept | 0.013 | -0.278 | 0.304 | 0.559 |
| A+ | [^18^F]MK6240 | Time | 0.473 | 0.254 | 0.693 | < 0.001 |
| A+ | [^18^F]MK6240 | APOE ɛ4 carriership | -0.028 | -0.359 | 0.303 | 0.838 |
| A+ | [^18^F]MK6240 | Age | -0.088 | -0.265 | 0.09 | 0.332 |
| A+ | [^18^F]MK6240 | Male sex | -0.012 | -0.328 | 0.304 | 0.938 |
| A+ | [^18^F]MK6240 | Baseline | 0.077 | -0.104 | 0.258 | 0.401 |
| A+ | [^18^F]MK6240 | Time:APOE ɛ4 carriership | -0.074 | -0.367 | 0.218 | 0.616 |

**Table S4. Demographics of all participants with longitudinal data in ADNI.**

Abbreviations: CI = Cognitively impaired, E4- = APOE ɛ4 non-carriers, E4+ = APOE ɛ4 carriers, SD = Standard deviation, SUVR = Standard uptake value ratio.

| Group | A- E4- | A- E4+ | A+ E4- | A+ E4+ |
| --- | --- | --- | --- | --- |
| N (%) | 328 (39.57) | 85 (10.25) | 162 (19.54) | 254 (30.64) |
| Follow-up, years, mean (SD) | 3.99 (2.39) | 3.45 (2.13) | 3.76 (2.27) | 3.48 (2.08) |
| Female, N (%) | 155 (47.26) | 32 (37.65) | 73 (45.06) | 114 (44.88) |
| Cognitively unimpaired, N (%) | 170 (51.83) | 42 (49.41) | 66 (40.74) | 47 (18.5) |
| Mild cognitive impairment, N (%) | 139 (42.38) | 41 (48.24) | 74 (45.68) | 143 (56.3) |
| Alzheimer's dementia, N (%) | 11 (3.35) | 1 (1.18) | 17 (10.49) | 56 (22.05) |
| Other neurological disease, N (%) | 8 (2.44) | 1 (1.18) | 5 (3.09) | 8 (3.15) |
| APOE ɛ4 homozygous, N, (%) | 0 (0) | 8 (9.41) | 0 (0) | 56 (22.05) |
| Age, mean (SD) | 72.4 (7.07) | 68.62 (7.42) | 74.96 (6.53) | 72.37 (6.42) |
| MMSE, mean (SD) | 28.66 (2.06) | 28.72 (1.38) | 27.69 (2.4) | 26.32 (3.91) |
| [^18^F]Florbetapir, SUVR, mean (SD) | 1 (0.05) | 1.02 (0.05) | 1.33 (0.19) | 1.38 (0.17) |
| [^18^F]AV1451, SUVR, mean (SD) | 1.24 (0.13) | 1.19 (0.11) | 1.55 (0.62) | 1.77 (0.57) |
| CSF Aβ1-42, pg/mL, mean (SD) | 1414.48 (319.07) | 1273 (386.54) | 896.45 (385.94) | 680.51 (253.83) |
| p-tau217, pg/mL, mean (SD) | 0.2 (0.25) | 0.16 (0.15) | 0.39 (0.3) | 0.51 (0.4) |
| p-tau181, pg/mL, mean (SD) | 14.97 (12.74) | 15.8 (11.72) | 18.91 (10.19) | 22.43 (9.99) |

**Table S5. Number of longitudinal ADNI participants per timepoint.**

| Amyloid status | A- | A- | A- | A- | A+ | A+ | A+ | A+ |
| --- | --- | --- | --- | --- | --- | --- | --- | --- |
| Year | p-tau217 | p-tau181 | [^18^F]Florbetapir | [^18^F]AV1451 | p-tau217 | p-tau181 | [^18^F]Florbetapir | [^18^F]AV1451 |
| 0 | 8 | 265 | 282 | 64 | 19 | 258 | 282 | 70 |
| 1 |  | 7 | 2 | 5 |  | 1 |  | 2 |
| 1.5 |  |  |  |  |  | 1 | 1 |  |
| 2 | 2 | 253 | 255 | 34 | 6 | 258 | 266 | 65 |
| 2.5 |  | 1 | 1 |  |  | 1 | 3 |  |
| 3 |  | 7 | 5 |  | 3 | 6 | 7 |  |
| 3.5 |  |  |  |  |  | 2 | 2 |  |
| 4 | 4 | 115 | 137 | 38 | 3 | 94 | 104 | 18 |
| 4.5 |  | 7 | 11 | 4 | 1 | 4 | 5 | 2 |
| 5 |  | 21 | 21 |  | 3 | 22 | 23 |  |
| 5.5 |  | 2 | 3 |  |  | 4 | 9 |  |
| 6 |  | 20 | 22 |  |  | 29 | 28 |  |
| 6.5 |  | 4 | 7 |  | 1 | 2 | 4 |  |
| 7 |  | 23 | 24 |  |  | 23 | 23 |  |
| 7.5 |  |  | 1 |  | 1 | 3 | 3 |  |
| 8 |  | 15 | 15 |  |  | 18 | 16 |  |
| 8.5 |  | 1 | 2 |  |  |  | 1 |  |
| 9 |  | 6 | 7 |  |  | 9 | 9 |  |
| 9.5 |  | 1 | 2 |  |  | 2 | 2 |  |
| 10 |  |  | 1 |  | 1 | 1 | 2 |  |
| 10.5 |  |  |  |  |  |  | 1 |  |
| 11 | 1 |  | 1 |  | 1 |  | 2 |  |
| 11.5 | 1 |  | 1 |  |  |  |  |  |
| 12 |  |  | 2 |  |  |  |  |  |
| 13.5 |  |  |  |  | 1 |  | 1 |  |

**Table S6. Results of longitudinal amyloid and tau PET measurements in A- and A+ APOE ɛ4 carriers and non-carriers in ADNI.**

Abbreviations: CI = Confidence interval, p-tau = phospho-tau, St. β = Standardized β-estimate.

| Amyloid status | Biomarker | Parameter | St. β | 95% CI down | 95% CI up | *P*-value |
| --- | --- | --- | --- | --- | --- | --- |
| A- | p-tau181 | Intercept | -0.029 | -0.124 | 0.067 | 0.766 |
| A- | p-tau181 | Time | -0.012 | -0.08 | 0.056 | 0.729 |
| A- | p-tau181 | APOE ɛ4 carriership | 0.003 | -0.167 | 0.173 | 0.793 |
| A- | p-tau181 | Age | 0.069 | 0.004 | 0.134 | 0.038 |
| A- | p-tau181 | Male sex | 0.051 | -0.075 | 0.177 | 0.425 |
| A- | p-tau181 | Baseline | -0.512 | -0.575 | -0.449 | < 0.001 |
| A- | p-tau181 | Time:APOE ɛ4 carriership | -0.029 | -0.202 | 0.144 | 0.746 |
| A- | [^18^F]Florbetapir | Intercept | 0 | -0.111 | 0.11 | < 0.001 |
| A- | [^18^F]Florbetapir | Time | -0.04 | -0.115 | 0.035 | 0.297 |
| A- | [^18^F]Florbetapir | APOE ɛ4 carriership | 0.175 | -0.017 | 0.366 | 0.983 |
| A- | [^18^F]Florbetapir | Age | -0.055 | -0.13 | 0.019 | 0.146 |
| A- | [^18^F]Florbetapir | Male sex | -0.045 | -0.191 | 0.101 | 0.544 |
| A- | [^18^F]Florbetapir | Baseline | -0.338 | -0.413 | -0.264 | < 0.001 |
| A- | [^18^F]Florbetapir | Time:APOE ɛ4 carriership | 0.184 | -0.009 | 0.376 | 0.081 |
| A- | [^18^F]AV1451 | Intercept | 0.017 | -0.294 | 0.327 | < 0.001 |
| A- | [^18^F]AV1451 | Time | 0.373 | 0.19 | 0.556 | < 0.001 |
| A- | [^18^F]AV1451 | APOE ɛ4 carriership | -0.477 | -1.021 | 0.066 | 0.062 |
| A- | [^18^F]AV1451 | Age | -0.224 | -0.506 | 0.058 | 0.12 |
| A- | [^18^F]AV1451 | Male sex | 0.2 | -0.276 | 0.675 | 0.407 |
| A- | [^18^F]AV1451 | Baseline | -0.326 | -0.584 | -0.068 | 0.016 |
| A- | [^18^F]AV1451 | Time:APOE ɛ4 carriership | 0.22 | -0.261 | 0.702 | 0.364 |
| A+ | [^18^F]Florbetapir | Intercept | 0.009 | -0.125 | 0.144 | 0.048 |
| A+ | [^18^F]Florbetapir | Time | 0.201 | 0.095 | 0.307 | < 0.001 |
| A+ | [^18^F]Florbetapir | APOE ɛ4 carriership | -0.032 | -0.184 | 0.12 | 0.691 |
| A+ | [^18^F]Florbetapir | Age | 0.007 | -0.067 | 0.082 | 0.846 |
| A+ | [^18^F]Florbetapir | Male sex | 0.014 | -0.13 | 0.158 | 0.846 |
| A+ | [^18^F]Florbetapir | Baseline | -0.127 | -0.2 | -0.054 | 0.001 |
| A+ | [^18^F]Florbetapir | Time:APOE ɛ4 carriership | -0.076 | -0.219 | 0.066 | 0.293 |
| A+ | [^18^F]AV1451 | Intercept | -0.099 | -0.435 | 0.237 | 0.652 |
| A+ | [^18^F]AV1451 | Time | 0.34 | 0.061 | 0.62 | 0.018 |
| A+ | [^18^F]AV1451 | APOE ɛ4 carriership | 0.114 | -0.279 | 0.507 | 0.74 |
| A+ | [^18^F]AV1451 | Age | -0.144 | -0.359 | 0.07 | 0.185 |
| A+ | [^18^F]AV1451 | Male sex | 0.088 | -0.344 | 0.52 | 0.687 |
| A+ | [^18^F]AV1451 | Baseline | 0.357 | 0.159 | 0.555 | 0.001 |
| A+ | [^18^F]AV1451 | Time:APOE ɛ4 carriership | 0.216 | -0.165 | 0.597 | 0.262 |

**
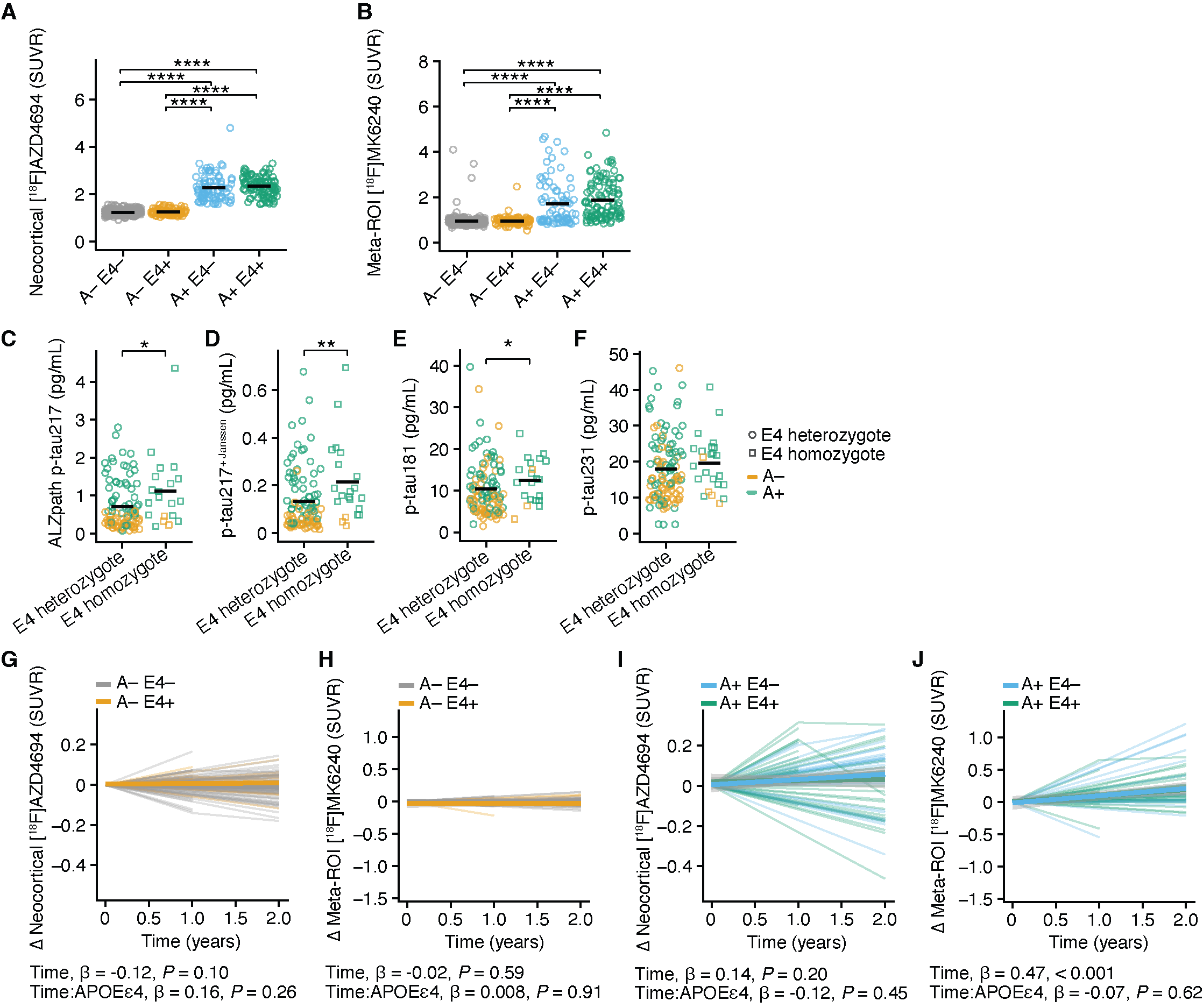
Figure S1. APOE ɛ4 do not have differences in PET-measured tau and Aβ accumulation in TRIAD.** (**A-B**) Neocortical [^18^F]AZD4696 standard uptake value ratio (SUVR) (**A**) and meta-ROI [^18^F]MK6240 SUVR (**B**) at the first visit in people with (A+) and without (A-) Aβ accumulation who are APOE ɛ4 carriers (A- E4+, *n* = 92; A+ E4+, *n* = 110) or non-carriers (A- E4- *n* = 260; A+ E4- *n* = 82). Unpaired FDR-adjusted *t*-tests were used for statistical comparisons. *****P* < 0.0001. (**C–F**) Plasma ALZpath p-tau217 (C), p-tau217^+ Janssen^ (D), p-tau181 (E) and p-tau231 (F; all in pg/mL) in APOE ɛ4 heterozygous (ALZpath p-tau217, *n* = 94; p-tau217^+ Janssen^, *n* = 100; p-tau181, *n* = 107; p-tau231, *n* = 113) and homozygous carriers (ALZpath p-tau217, *n* = 20; p-tau217^+ Janssen^, *n* = 21; p-tau181, *n* = 21; p-tau231, *n* = 24). Non-parametric Mann-Whitney-U test was performed. **P* < 0.05, ***P* < 0.01. (**G-H**) Longitudinal changes of neocortical [^18^F]AZD4696 SUVR (**G**) and meta-ROI [^18^F]MK6240 SUVR (**H**) in participants without Aβ accumulation who are APOE ɛ4 non-carriers (A- E4-; [^18^F]AZD4696, *n* = 85; [^18^F]MK6240 SUVR, *n* = 80) or carriers (A- E4+; [^18^F]AZD4696, *n* = 29; [^18^F]MK6240 SUVR, *n* = 27). (**I-J**) Longitudinal changes of neocortical [^18^F]AZD4696 SUVR (**I**) and meta-ROI [^18^F]MK6240 SUVR (**J**) in participants with Aβ accumulation who are APOE ɛ4 non-carriers (A+ E4-; [^18^F]AZD4696, *n* = 36; [^18^F]MK6240 SUVR, *n* = 31) or carriers (A+ E4+; [^18^F]AZD4696, *n* = 42; [^18^F]MK6240 SUVR, *n* = 45). Linear mixed effects models were used for statistical analyses. The standardized β-estimates and *P*-values for the time and interaction between time and APOE ɛ4 carriership is provided in the figure. The complete statistical results are shown in the Table S3.

**
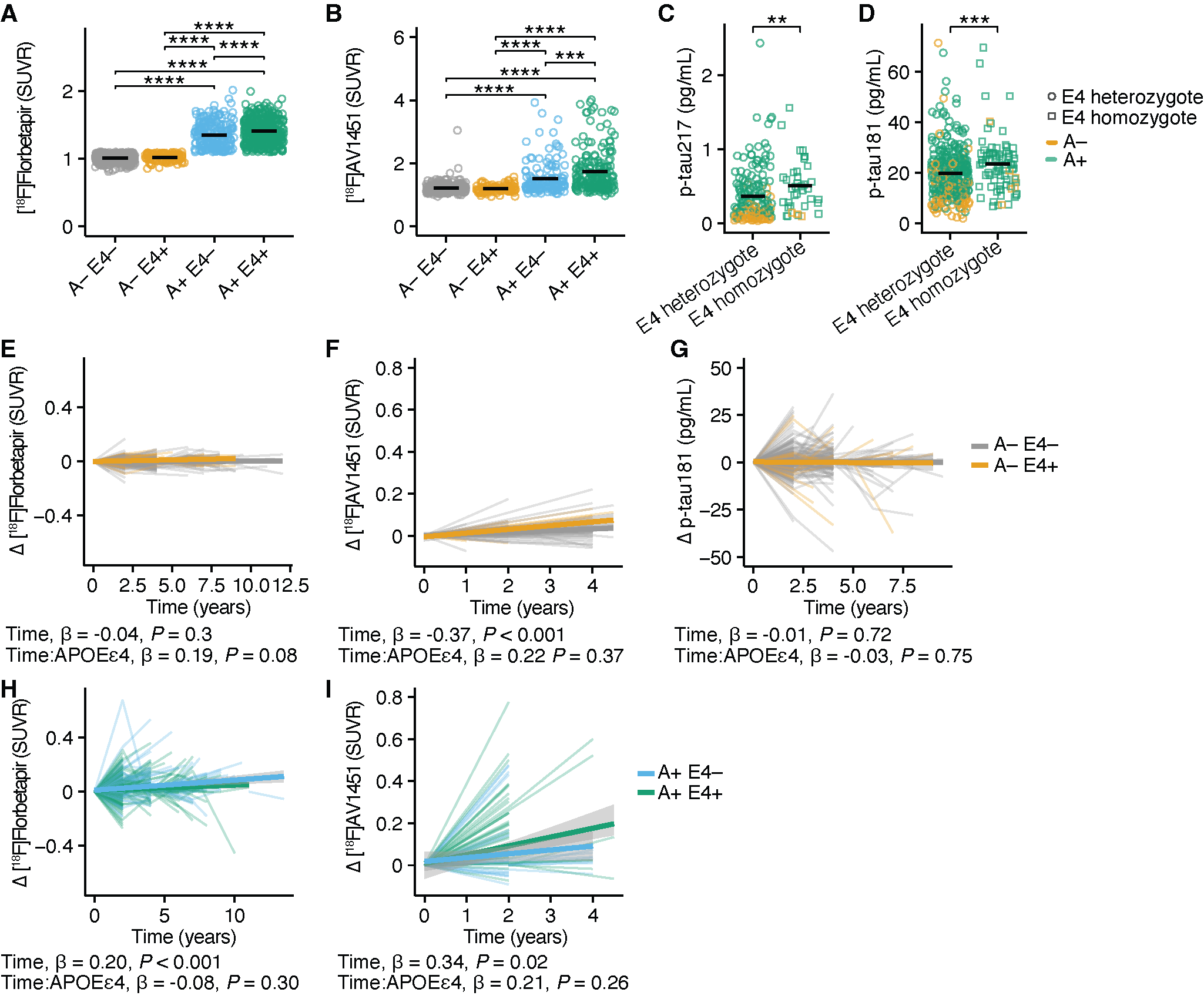
**

**Figure S2. APOE ɛ4 do not have differences in PET-measured tau and Aβ accumulation in ADNI.** (**A-B**) Neocortical [^18^F]Florbetapir standard uptake value ratio (SUVR) (**A**) and meta-ROI [^18^F]AV1451 SUVR (**B**) at the first visit in people with (A+) and without (A-) Aβ accumulation who are APOE ɛ4 non-carriers ([^18^F]Florbetapir, A- E4- *n* = 393; A+ E4- *n* = 213; [^18^F]AV1451, A- E4- *n* = 314; A+ E4- *n* = 133) or carriers ([^18^F]Florbetapir, A- E4+, *n* = 111; A+ E4+, *n* = 368; [^18^F]AV1451, A- E4+, *n* = 99; A+ E4+, *n* = 186) Unpaired FDR-adjusted *t*-tests were used for statistical comparisons. *****P* < 0.0001. (**C–D**) Plasma p-tau217 (C), p-tau181 (D) in APOE ɛ4 heterozygous (p-tau217, *n* = 166; p-tau181, *n* = 311) and homozygous carriers (p-tau217, *n* = 38; p-tau181, *n* = 80). Non-parametric Mann-Whitney-U test was performed. ***P* < 0.01, ****P* < 0.001. (**E-G**) Longitudinal changes of neocortical [^18^F]Florbetapir SUVR (**E**), meta-ROI [^18^F]AV1451 SUVR (**F**), and plasma p-tau181 (**G**) in participants without Aβ accumulation who are APOE ɛ4 non-carriers (A- E4-; [^18^F]Florbetapir, *n* = 272; [^18^F]AV1451 SUVR, *n* = 39; p-tau181, *n* = 264) or carriers (A- E4+; [^18^F]Florbetapir, *n* = 60; [^18^F]AV1451 SUVR, *n* = 11; p-tau181, *n* = 57). (**H-I**) Longitudinal changes of neocortical [^18^F]Florbetapir SUVR (**H**), and meta-ROI [^18^F]AV1451 SUVR (**I**) in participants with Aβ accumulation who are APOE ɛ4 non-carriers (A+ E4-; [^18^F]Florbetapir, *n* = 135; [^18^F]AV1451 SUVR, *n* = 24) or carriers (A+ E4+; [^18^F]Florbetapir, *n* = 214; [^18^F]AV1451 SUVR, *n* = 35). Linear mixed effects models were used for statistical analyses. The standardized β-estimates and *P*-values for the time and interaction between time and APOE ɛ4 carriership is provided in the figure. The complete statistical results are shown in the Table S6.
